# Cortical Thickness Adaptive Response to Mechanical Loading Depends on Periosteal Position and Varies Linearly With Loading Magnitude

**DOI:** 10.3389/fbioe.2021.671606

**Published:** 2021-06-18

**Authors:** Corey J. Miller, Silvia Trichilo, Edmund Pickering, Saulo Martelli, Peter Delisser, Lee B. Meakin, Peter Pivonka

**Affiliations:** ^1^School of Mechanical, Medical and Process Engineering, Queensland University of Technology, Brisbane, QLD, Australia; ^2^St. Vincent’s Department of Surgery, University of Melbourne, Melbourne, VIC, Australia; ^3^School of Veterinary Sciences, University of Bristol, Bristol, United Kingdom

**Keywords:** cortical bone, adaptation, mechanical loading, local adaptation, cortical thickness, periosteal apposition, tibia loading, mouse model

## Abstract

The aim of the current study was to quantify the local effect of mechanical loading on cortical bone formation response at the periosteal surface using previously obtained μCT data from a mouse tibia mechanical loading study. A novel image analysis algorithm was developed to quantify local cortical thickness changes (ΔCt.Th) along the periosteal surface due to different peak loads (0N ≤ F ≤ 12N) applied to right-neurectomised mature female C57BL/6 mice. Furthermore, beam analysis was performed to analyse the local strain distribution including regions of tensile, compressive, and low strain magnitudes. Student’s paired *t*-test showed that ΔCt.Th in the proximal (25%), proximal/middle (37%), and middle (50%) cross-sections (along the z-axis of tibia) is strongly associated with the peak applied loads. These changes are significant in a majority of periosteal positions, in particular those experiencing high compressive or tensile strains. No association between F and ΔCt.Th was found in regions around the neutral axis. For the most distal cross-section (75%), the association of loading magnitude and ΔCt.Th was not as pronounced as the more proximal cross-sections. Also, bone formation responses along the periosteum did not occur in regions of highest compressive and tensile strains predicted by beam theory. This could be due to complex experimental loading conditions which were not explicitly accounted for in the mechanical analysis. Our results show that the bone formation response depends on the load magnitude and the periosteal position. Bone resorption due to the neurectomy of the loaded tibia occurs throughout the entire cross-sectional region for all investigated cortical sections 25, 37, 50, and 75%. For peak applied loads higher than 4 N, compressive and tensile regions show bone formation; however, regions around the neutral axis show constant resorption. The 50% cross-section showed the most regular ΔCt.Th response with increased loading when compared to 25 and 37% cross-sections. Relative thickness gains of approximately 70, 60, and 55% were observed for F = 12 N in the 25, 37, and 50% cross-sections. ΔCt.Th at selected points of the periosteum follow a linear response with increased peak load; no lazy zone was observed at these positions.

## Introduction

Osteoporosis is a degenerative bone disease characterised by long-term bone loss and fragility (Black and Rosen, 2016). To counter osteoporosis, several drugs have been developed to either reduce or reverse the bone loss process. Despite the success in reducing the degeneration of osteoporosis, drug treatments can have significant side effects, and the positive effect on the bone mass is often lost upon discontinuation of the drug dosing regimen (McClung, 2016; Minisola et al., 2019). Pharmacologically, drug treatments such as PTH are generalised therapies and do not target specific bones. Exercise, on the other hand, has been identified as a safe alternative to restore bone mass (Bliuc et al., 2013; Ebeling et al., 2013; Beck et al., 2017); mechanical loading interventions can act as a potent anabolic stimulus with the ability to strategically restore bone mass in regions of bone that undergo significant loading, both in animal models and humans (Ozcivici et al., 2010).

Bone tissue adapts its mass and structure to the habitual mechanical loading environment (Rubin and Lanyon, 1985; Ozcivici et al., 2010; Pivonka et al., 2018). Several animal loading models have been developed to investigate the relations between the applied mechanical load, the changes in bone mass, and the bone cells involved in mechano-transduction (Meakin et al., 2014; Javaheri et al., 2019). Among these models, the mouse tibia loading model is commonly used to assess both trabecular and cortical bone adaptation responses (De Souza et al., 2005; Sugiyama et al., 2010, 2012). In this model, the tibia is subjected to cyclic, compressive load, while the contralateral tibia serves as an internal control. Common metrics used to assess bone adaptation to mechanical loading consider global morphological variations at either the entire bone level (e.g., bone volume change), or at the entire bone slice level (e.g., cross-sectional area change, moment of area change). A comprehensive study by Sugiyama et al., 2012 (Sugiyama et al., 2012) explored the influence of peak dynamic load on bone adaptation. The primary focus was to analyse the effect of peak dynamic loads (ranging from 0 to 14 N) on changes in cortical area (ΔCt.Ar), determined through μCT endpoint imaging. They concluded that changes in cortical bone cross-sectional area are linearly related to the peak applied load. However, bone adaption is a local (i.e., site-specific) phenomenon governed by the local strain (Fritton et al., 2005; Razi et al., 2015). As such, metrics operating on the entire bone or on a slice level are unable to provide detailed insights into a load-adaptation response law. While the study of Sugiyama et al. did observe site-specific adaptation, the load-adaptation response was not explored this in a quantitative manner. A more detailed evaluation of bone’s adaptive response to local strain can be obtained by analysing the local cortical thickness change (ΔCt.Th).

To this end, several studies have explored the local cortical thickness variation (ΔCt.Th) (Halloran et al., 2002; Stadelmann et al., 2011; Sugiyama et al., 2012; Galea et al., 2015; Birkhold et al., 2016; Roberts et al., 2020), commonly using a minimum distance metric (i.e., the shortest distance between periosteal and endosteal surfaces) (Hildebrand and Rüegsegger, 1997; Bouxsein et al., 2010). Pereira et al. (2015) used the same method to analyse ΔCt.Th but instead considered spatially discrete locations, reporting ΔCt.Th in a polar coordinate system around the centroid. This technique provided promising results for the majority of the tibial cross-sections analysed; however, it is inadequate for bony protrusions such as the tibial ridge. Furthermore, while new tissue forms normal to the bone surface (Graham et al., 2012; Pereira et al., 2015; Zhang et al., 2019), the use of a minimum distance technique does not accurately capture ΔCt.Th when the direction of adaptation (i.e., normal to the surface) is highly offset from the radial direction. Similarly, radial coordinates create issues when determining periosteal and endosteal edges, where in some cases up to four cortical intersection points can be identified for a given radial direction (Bab et al., 2007).

To account for the irregular shape of the mouse tibia, this study proposes a new technique for measuring cortical thickness variations. A novel image post-processing algorithm was developed to allow the calculation of the local ΔCt.Th around the perimeter of the tibia using a combined minimum distance and normal distance approach. The experimental results of Sugiyama et al. (2012) were re-analysed to quantify local cortical thickness changes and their association to the peak load applied. The analysis was conducted for four commonly studied cross-sections in the mouse tibia loading model (i.e., 25, 37, 50, 75%). Furthermore, mechanical analysis using beam theory was performed in order to relate the obtained cortical thickness changes to the local mechanical loading environment and identify regions of high and low strains, respectively.

## Materials and Methods

The endpoint imaging data used in this study was previously reported by Sugiyama et al. (2012). As such, we have provided a brief summary of the experimental design and imaging process here; for a more complete description see (Sugiyama et al., 2012). Following this, a detailed description of the newly developed image processing algorithm used to extract local Δ Ct.Th measurements of tibial cross sections at selected regions is presented.

### Experimental Design

A total of 48 female C57BL/6 mice were divided evenly into eight groups, with each group assigned to one of eight peak load magnitudes (F = 0, 2, 4, 6, 8, 10, 12 or 14 N) (Sugiyama et al., 2012). For the purpose of our study, the F = 14 N loading case was excluded due to the formation of woven bone in several animals. Each mouse was subjected to a right sciatic neurectomy at 17 weeks of age, in order to minimise the natural loading in their right tibiae (i.e., muscle contraction forces) and simulate a condition of mechanical disuse. From day 5 after neurectomy, every second day, and for two weeks, the right tibia of each mouse was subjected to external mechanical loading. A non-invasive servo hydraulic loading machine applied 40 cycles of intermittent loading, with each cycle consisting of: (i) 0.5 N static preload, (ii) 500 N/s ramp up to target peak load, (iii) a 0.05 second hold at peak load, (iv) −500 N/s ramp down to static preload, (v) 10 s rest interval. This has been shown to significantly stimulate loading-related bone gain (Rubin and Lanyon, 1984; Fritton and Rubin, 2001; Robling et al., 2001; Srinivasan et al., 2002; De Souza et al., 2005; Sugiyama et al., 2010, 2011; Moustafa et al., 2012). The left tibia of each mouse was used as contralateral control (Sugiyama et al., 2010; McKenzie and Silva, 2011). At day 21 after neurectomy, the mice were sacrificed, and both left and right tibiae were scanned using μCT imaging. Whole tibiae were imaged using the SkyScan 1172 (SkyScan, Kontich, Belgium) with an isotropic resolution of 4.78 μm. An X-ray voltage of 50 kV was applied, with 0.5 mm aluminium filtration. The scans were over 180 degrees with a 0.5-degree rotation step.

### Beam Theory Analysis of Tibia

The mechanical analysis presented in this paper aims to link the strains in the cortical cross section to the observed thickness changes. We assume that the tibia represents a slender beam structure and, consequently, can be analysed using Euler-Bernoulli beam theory (Hjelmstad, 2005; Bauchau and Craig, 2009; Buenzli et al., 2013; Lerebours et al., 2016; Trichilo, 2018; Ashrafi et al., 2020). The purpose of this analysis is not to provide a direct link between strain magnitudes and the adaptive response, but rather to identify compressive and tensile regions of strain and to observe general trends of strain magnitude across a given cross-section.

The load F was assumed to act on the tibial plateau in the *z*-direction between the tibial condyles; this location was previously suggested from strain gauge studies (Pickering et al., 2021). In a particular cross section (z), F induces a normal force (F = N) and bending moments *M*_*x*_ ( = *F*⋅*I*_*y*_) and *M*_*y*_ ( = *F*⋅*I*_*x*_), where *I*_*y*_ and *I*_*x*_ represents the distance of the load *F* to the *x* and *y* axis respectively. Knowing the internal beam quantities once can calculate the axial strain according to:

(1) $$\begin{array}{c} \varepsilon \left(x,y\right)=\frac{1}{E}\sigma \left(x,y\right)=\frac{1}{E}\left(\frac{N}{A}-\frac{M_{y}I_{x}+M_{x}I_{xy}}{I_{x}I_{y}-I_{xy}^{2}}x+\frac{M_{x}I_{y}+M_{y}I_{xy}}{I_{x}I_{y}-I_{xy}^{2}}y\right) \end{array}$$

where *I*_*x*_ and *I*_*y*_ are the second moments of area with respect to the *x*- and *y*-axis, respectively and *I*_*xy*_ is the product moment of area. In Eq. 1 bone was assumed to be a linear-elastic material with a Young’s modulus E = 17 GPa (Kohles et al., 1997). A maximum load of F = 12 N was applied to aid in differentiation of strain magnitudes, aligning with the peak load used in the experimental protocol.

To compute the second moments of area *I*_*x*_, *I*_*y*_, and *I*_*xy*_ of each cross-section, a customised algorithm to automatically segment μCT images was developed in MATLAB. μCT images were first binarised using Otsu’s thresholding method. A filter was then applied to the images to close any small holes in the tibial cross-section (representing blood vessel channels), and to smooth the tibial boundaries. The second moments of area were calculated using parallel axis theorem, treating each white pixel (i.e., bone) in the image as a square of area 22.84 μm^2^.

### Image Post-processing Algorithm

Each stack of tibial μCT images was normalised along the proximal-distal direction of the tibia (i.e., z-axis), with z = 0% referring to the most proximal slice and z = 100% referring to the most distal slice. In this study, the response to mechanical loading was analysed on a single cross-sectional slice taken from the z = 25% (proximal), z = 37% (proximal-middle), z = 50% (middle), and z = 75% (distal) locations of the tibia. Note that we also performed the following methodology over a representative stack of images spanning approx. 0.5 mm of the tibia (±0.25 mm from selected slice), as has been commonly done in previous works (see Sugiyama et al., 2012). A comparison of the results from using a single slice and the representative stack can be found in Supplementary Figure 1; measurements in a single slice were found to not differ significantly from the representative stack. The selected μCT images were grouped based on peak load applied, cross-section analysed and control/loaded tibia.

**FIGURE 1:**
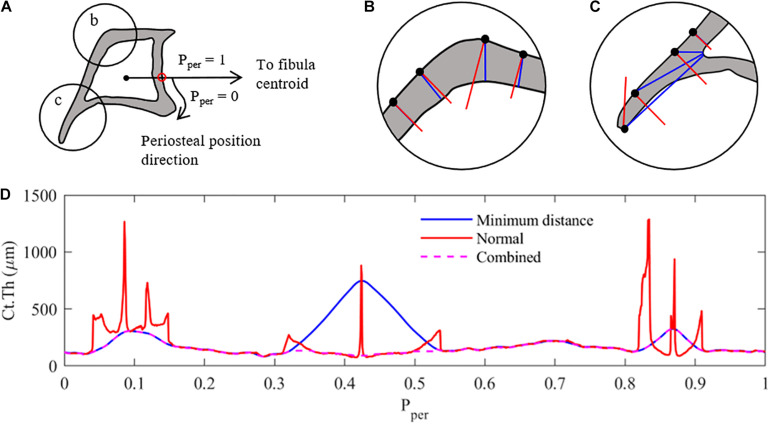
Measurement methodology used to analyse the cortical thickness of mice tibia (z = 25% section). **(A)** Pre-processing – the cortical boundaries are located (periosteum and endosteum), and a characteristic periosteal point is determined as the intersection of a line connecting the centroids of the tibia and fibula (red dot). The periosteal length (perimeter) is normalised between 0 and 1. Ct.Th measurements at each periosteal point are taken clockwise around the tibial perimeter. **(B)** Thickness measurement case 1: minimum distance method (red) and surface-normal blue) measurements around the cortex. Similar results with minimum distance providing shorter measurements. **(C)** Thickness measurement case 2: thickness measurements along the tibial ridge, showing major differences between measurement methods with tangent-normal providing shorter measurements. **(D)** Combination of the two measurement methods, selecting the smallest distance determined by either measurement method, to create the most representative cortical thickness distribution of the tibial image. Combined Results are then filter using a 2nd order Butterworth filter.

Following the binarisation process described above, pixels along the periosteal and endosteal envelopes were identified and mapped into an array. In order to compare the thickness along the periosteum between different limbs, periosteal position (P_*per*_) distributions were aligned across all tibiae at a given z cross-section through the location of a characteristic point (i.e., pixel) on the tibial periosteum (P_*per*_ = 0). This characteristic point was identified as the intersection between the tibia periosteum and the line connecting the tibia and fibula centroids (Figure 1A). Starting at P_*per*_ = 0 and following a clockwise direction, the pixels along the periosteal surface were re-arranged and normalised between 0 and 1. In the case of the distal cross-section (z = 75%) where the fibula is absent, a faux fibula centroid was projected onto the plane from the 50% section.

When defining the local cortical thickness two types of measurements were used, as shown in Figures 1B,C: (i) a minimum distance measurement (shown in blue) and (ii) a perpendicular distance measurement (shown in red). The minimum distance method measured the distance between each periosteal pixel and the nearest endosteal pixel. The normal method measured the distance from each periosteal pixel to the next cortical edge (endosteal or periosteal) along a line perpendicular to the periosteum. In cross-sectional regions with approximately constant curvature of periosteum and endosteum, both techniques provided a similar result, as shown in Figure 1B. The more distally located cross-sections (z = 50 and 75%) confirm this trend for the majority of the periosteal surface. However, in regions with large curvature changes such as the tibial ridge (i.e., z = 25 and 37%), a large discrepancy between the two measurement techniques was observed, as highlighted in Figure 1C. In order to generate a thickness measurement which best represented bone adaptation in these sections [i.e., normal to the surface (Graham et al., 2012)], both thickness measures were calculated for each periosteal pixel and the smaller of the two measurement values was used to define the representative local cortical thickness Ct.Th (Figure 1D). This result was then filtered using a 2nd order lowpass Butterworth filter to remove high-frequency noise due to the measurement combination technique.

Since the results across images were different in length due to the variability between animals and the adaptation process of the loaded limbs, each Ct.Th distribution was re-sampled so that P_*per*_ contained *n* = 750 periosteal points. A further consideration was made when comparing cortical thickness distributions of the loaded and control limbs. As mentioned, the loaded limb presented a longer periosteum due to the adaptation process, therefore, to ensure an accurate comparison of the same cortical regions, a further alignment step was required. For the approximately circular cross-sections (z = 50 and 75%), cross-covariance was used to circularly shift one of the two thickness signals, to maximise the alignment with the other one. In the cases of the z = 25 and 37% cross-sections, where the growth/resorption along the tibial ridge had a significant effect on the alignment of P_*per*_ points between loaded and control tibia, a customised re-sampling methodology was developed. Four common peaks and/or troughs in the Ct.Th measurement distribution were identified in all the limbs analysed that correlated with key bony features, e.g., the tip of the tibial ridge. Thickness measurements between these key-points were re-sampled based on a fixed number of points, resulting in an optimal and consistent alignment between the peaks for all slices at that particular cross-section. This process was repeated for each mouse limb (right and left tibiae). It should be noted that the thickness along the periosteum was measured for both right and left limbs starting from P_*per*_ = 0 and following a clockwise direction in the cross-section. To be able to make left vs. right comparisons, all the left limb signals needed to be reversed. The thickness distributions were then compared between the right and the left tibiae of a mouse at each cross-section (z), for all considered loading conditions. The relative change in cortical thickness (ΔCt.Th) for each periosteal point was calculated as:

(2) $$\text{Δ}Ct.Th\left(P_{per}\right)\left[\%\right]=\frac{Ct.Th{\left(P_{per}\right)}_{right}-Ct.Th{\left(P_{per}\right)}_{left}}{Ct.Th{\left(P_{per}\right)}_{left}}\cdot 100$$

where P_*per*_ identifies the periosteal position at which the cortical thickness is evaluated at the right and left tibia. Eq. 2 is an extension of the equation used for calculating cortical area changes (see Sugiyama et al., 2012 for details) with respect to considering localised cortical thickness changes.

### Statistical Analysis

Mean values ($\bar{\Delta \mathrm{Ct}.\text{Th}}$) and standard deviations (SD) of ΔCt.Th were calculated across the six specimens within a loading condition at each point P_*per*_. For simplicity of notation, the symbol ΔCt.Th will denote mean cortical thickness changes throughout the rest of the manuscript. The results were evaluated through Student’s paired *t*-tests for each loading condition per tibial cross section, considering the link between the local thickness change at each periosteal surface position and the mechanical loading. For this investigation, a *p*-value <0.05 was considered statistically significant.

## Results

For clarity, results will first be presented for the middle region (z = 50%) as this section consists of an approximately circular cross-section and embodies the greatest strains. Results for the remaining three sections will be presented thereafter.

**FIGURE 2:**
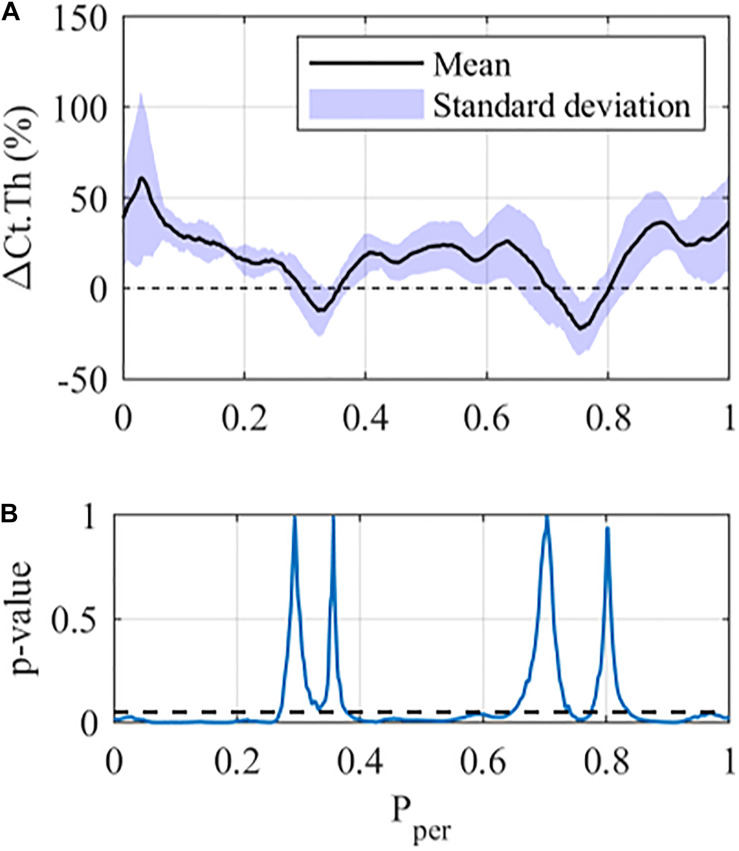
Local cortical thickness variation for the 12 N loading case at the middle tibial region (z = 50%): **(A)** Mean (black line) and standard deviation (Shaded area) along the periosteum ΔCt.Th ± SD vs P_*per*_ and **(B)** *p*-value using a student-paired *t*-test at each periosteal position (statistical significance indicated by values below the dashed line, i.e., *p* < 0.05).

Figure 2 shows the results of the Student’s paired t-tests for the 12 N loading case in the middle cross-section of the tibia. From Figure 2A one can see that the mean ΔCt.Th reaches values greater than 50% at selected positions along the periosteum (P_*per*_ ≈ 0.02), whereas other regions show zero or negative thickness change (0.3 < P_*per*_ < 0.35 and 0.7 < P_*per*_ < 0.80). Furthermore, mice within each loading group showed variable response to mechanical loading, with the standard deviation being approximately ±10% at the majority of periosteal surface. The results of the paired *t*-test indicate that local changes in cortical thickness were statistically significant (*p* < 0.05) over a large portion of the periosteal surface (Figure 2B), and the only regions demonstrating no significance were those with near zero cortical thickness change.

**FIGURE 3:**
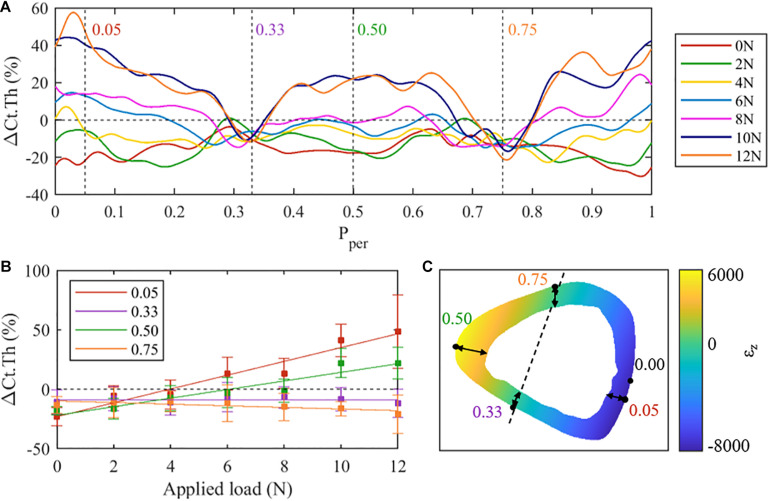
**(A)** Mean cortical thickness changes (ΔCt.Th) along the normalised periosteal position at the middle tibial cross-section (z = 50%) for all loading cases. Regions of interest have been identified at P_*per*_ = 0.05 (posterior surface), 0.33 (lateral), 0.50 (anterior), and 0.75 (medial). **(B)** Mean cortical thickness changes at selected regions of interest across all loading conditions. **(C)** Finite element results (F = 10 N) within the 50% cross-section.

Figure 3 shows the adaptation response along the periosteal surface in the z = 50% section for all peak loads investigated. For low peak loads (i.e., 4 N and lower), resorption was observed at most locations around the periosteal surface (negative ΔCt.Th, Figure 3A). At peak loads of 6 N and higher, positive ΔCt.Th (i.e., bone formation) begins to show along the periosteum. The region on the periosteum with bone formation increases with the increasing peak load. Note that the ΔCt.Th vs. F response is quasi linear, while the slope of the response depends on the periosteal position.

At approximately P_*per*_ = 0.33 and P_*per*_ = 0.75, consistent resorption was observed with little to no dependence on the applied load. In contrast, periosteal locations at approximately P_*per*_ = 0.05 and P_*per*_ = 0.5 show a large dependence on load magnitude, exhibiting near maximum changes in ΔCt.Th. To explore the load dependency further, ΔCt.Th has been reported in Figure 3B as a function of applied load for the four periosteal locations identified above, i.e. P_*per*_ = 0.05, 0.33, 0.50, 0.75. At locations P_*per*_ = 0.33 and P_*per*_ = 0.75 a nearly constant reduction of ΔCt.Th, independent of the magnitude of the applied peak load, is noted. At locations P_*per*_ = 0.05 and P_*per*_ = 0.50, a quasi-linear relationship between load magnitude and ΔCt.Th is observed. The P_*per*_ = 0.05 location (i.e., posterior-lateral surface) was noted to experience greater ΔCt.Th when compared to P_*per*_ = 0.50 (i.e., anterior-medial surface).

To test if there is a correlation between the thickness change along the periosteum and the axial strain (ε_*z*_) encountered in the cross section, beam theory was used to calculate ε_*z*_ in the middle cross-section (Figure 3C). Comparing the ΔCt.Th distribution (Figure 3A) and the axial strain (Figure 3C), apparent trends of the load-adaptation response can be observed; to investigate this further, four locations around the periosteum [posterior (P_*per*_ = 0.05), lateral (P_*per*_ = 0.33), anterior (P_*per*_ = 0.5), medial (P_*per*_ = 0.75)] were extracted and explored in Figure 3B. The posterior region experienced a higher strain magnitude compared to the anterior region (−8,198 and 6,075 με, respectively), coinciding with a higher ΔCt.Th along the periosteum in the same region. Likewise, the load independent locations on the periosteum (P_*per*_ = 0.33 and P_*per*_ = 0.75) appear to coincide with near zero axial strain. This region is commonly referred to as the neutral bending axis or neutral axis.

**FIGURE 4:**
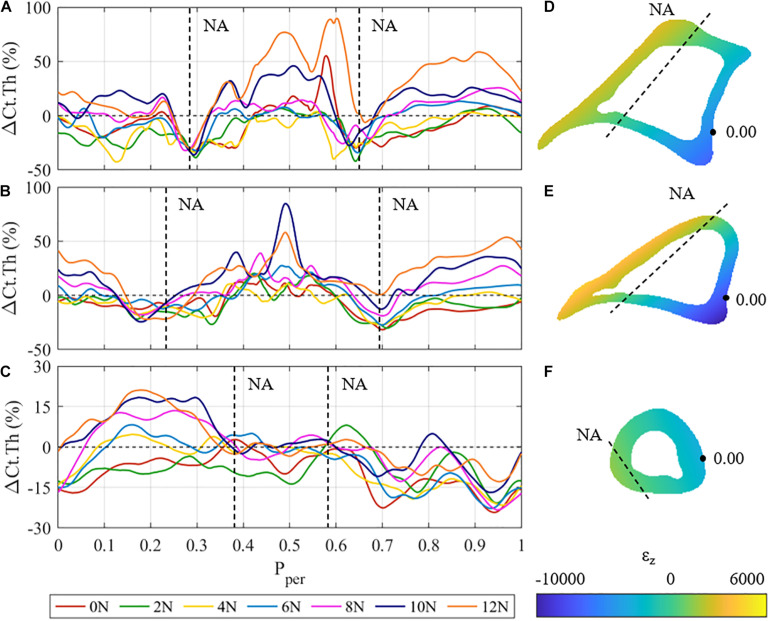
**(A–C)** Mean cortical thickness changes (ΔCt.Th) vs. normalised periosteal position (P_*per*_) at the: **(A)** proximal, **(B)** proximal-middle, and **(C)** distal tibial cross-section for all different loading cases. Vertical dashed lines represent the approximate P_*per*_ position of the expected neutral axis (NA). **(D–F)** Beam theory results for the: **(D)** proximal, **(E)** proximal-middle, and **(F)** distal tibial cross-sections. Dashed lines represent the approximate physical locations of the neutral axis (ε = 0).

Figure 4 shows the mean cortical thickness changes, i.e., ΔCt.Th curves of the proximal (z = 25%), proximal-middle (z = 37%), and distal (z = 75%) cross-sections of the tibia, as well as the respective strain distribution ε_z_ generated by a 12 N load for the corresponding cross sections. The proximal and proximal-middle cross-sections show similar trends in ΔCt.Th to the middle region discussed above (Figures 4A,B), with bone gain or loss responses occurring at different periosteal positions. In the proximal cross-section, the maximum bone formation response occurs in the tensile region of the cross-section (0.35 < P_*per*_ < 0.65). Cortical growth response to compressive loading produced up to a 55% increase in cortical thickness at P_*per*_ = 0.9 for 12 N peak load. The proximal-middle cross-section follows similar trends to the proximal one. Bone gain is higher in the tensile region (maximum change of 80% at P_*per*_ = 0.49 for 10 N peak load) than it is in compression (maximum 53% at P_*per*_ = 0.96 for 12 N peak load).

In both the proximal and proximal-middle cross-sections, bone resorption was observed around the medial neutral axis under all loading conditions (0.25 < P_*per*_ < 0.33 in the proximal region, 0.15 < P_*per*_ < 0.3 in the proximal-middle region). The lateral side of the neutral axis was observed to show smaller rates of resorption with increased load (0.6 < P_*per*_ < 0.7 in the proximal region, 0.65 < P_*per*_ < 0.75 in the proximal-middle region). Under the 12 N load, the proximal-middle region shows no loss of bone at the lateral side of the neutral axis.

The adaptive response in the distal cross-section of the tibia differs significantly from the other three regions (Figure 4C). Bone gains are the lowest of all the four investigated cross-sections, showing a maximum cortical thickness increase of 21% at P_*per*_ = 0.17. The anterior surface (0.35 < P_*per*_ < 0.65) shows that the cortical surface remains relatively stable with zero change to cortical surface for F ≥ 4 N. Significant bone gain was observed in the posteromedial section of the cross-section (0 ≤ P_*per*_ < 0.35) for F ≥ 6 N, whereas the posterolateral section experienced resorption across all loading conditions. The neutral axis of the distal region cannot be clearly identified from the obtained thickness results.

The results of the beam analysis revealed peak tensile strains of 2,583, 4,187, and 964 με at P_*per*_ ≈ 0.5 for the proximal (Figure 4D), proximal-middle (Figure 4E), and distal (Figure 4F) cross-sections, respectively. Peak compressive strains were −6,875, −9,994, and −3,381 με at P_*per*_ ≈ 0.05. This is consistent with the middle region, where higher strain magnitudes were observed on the posterior side when compared to the anterior. It should also be noted that the majority of the distal cross section is under compressive loading with only a small tensile region (Figure 4F).

## Discussion

Bone adaptation is a local phenomenon. This has been demonstrated previously along the axis of the tibia or in discrete segments of the tibia (Sugiyama et al., 2010; Lu et al., 2016; Galea et al., 2020; Roberts et al., 2020; Scheuren et al., 2020). However, this work is the first to demonstrate the link between magnitude of axial load and adaptive response around the periosteum of the tibia at a cross-section level. Our results clearly show that the cortical thickness change around the periosteal surface varies linearly with loading magnitude. Furthermore, the slope of this adaptive response depends on the periosteal position.

Quantifying the adaptive response through ΔCt.Th provides important insights into understanding the localised changes compared to other metrics (e.g., ΔCt.Ar). Shown in Figure 3B, the bone’s adaptive response was found to have a quasi-linear relationship between load magnitude and bone formation. This observation supports previous findings, such as those of Sugiyama et al. (2012) who found ΔCt.Ar increased linearly with the applied load. However, the use of such metrics (i.e., non-localised) mask the true magnitude of adaptation. Under a peak load of 12 N at the 50% section, Sugiyama et al. (2012) recorded an average ΔCt.Ar of 15.5 ± 2.1% (Sugiyama et al., 2012). However, for the same load, at the same section, we show that ΔCt.Th can vary between −20 and +60%; this suggests that broad metrics such as ΔCt.Ar are insufficient to fully describe the adaptation response to mechanical stimuli.

Many studies have shown a link between local adaptive response and local strain magnitude (Fyhrie and Carter, 1986; Robling et al., 2006; Webster et al., 2012; Schulte et al., 2013; Lambers et al., 2015; Pereira et al., 2015; Carriero et al., 2018; Tiwari et al., 2018). However, none of these studies have looked at the effect of different loading magnitudes on the observed local changes. Here we compared the local adaptive response as a function of loading magnitude in different cortical cross-sections. Focusing on the 50% cross-section, the posterior surface (P_*per*_ = 0.05) experienced larger bone formation as a function of peak load (i.e., larger slope of the F vs. ΔCt.Th curve) compared to the anterior surface (P_*per*_ = 0.5), as shown in Figures 3A,B. The increased response in this region correlates to the strain magnitude resulting from combined bending and axial load. The peak compressive strain is greater than the peak tensile strain. As such, the compressive surface experiences a larger bone formation response. The same trend is seen in the 25 and 37% cross sections, shown in Figures 4A,B.

Regions of low strain (i.e., those near the neutral axis) experienced bone resorption. In the 50% cross-section, the medial and lateral surfaces (P_*per*_ ≈ 0.75, 0.33, respectively) experienced resorption, independently of the load applied. This finding stands in contrast to the study performed by Pereira et al. (2015). Most notably, in non-neurectomised C57BL/6 mice, adaptation to loading of F = 13 N was all positive, i.e., no bone resorption occurred (Pereira et al., 2015; Trichilo, 2018). One explanation for the observed bone loss in the neurectomised mice is the fact that muscle action comprises a significant portion of the habitual strain state. Without the continual influence of muscle activation to maintain mechanical homeostasis at the neutral axis, these regions will undergo bone resorption to readjust to their new habitual state.

This same trend of resorption around the neutral axis is observed in the 25 and 37% sections. In the case of the 75% cross-section, however, a more general trend of resorption is observed, with up to half of the cross-section experiencing resorption under the highest peak loads. Strains in this cross-section were noticeably smaller than the strains in the other three. This might be related to this cross-section of the tibia being aligned with the longitudinal axis, resulting in low bending moments, and thus axial compression dominates.

The bone adaptive response observed in Figures 3A, 4A,B can be further viewed through the lens of beam theory and second moment of area. A higher second moment of area leads to lower overall strain; the most efficient way to increase the second moment of area is by adding new material furthest away from the neutral axis. In doing so, bone maximises its strength while optimising the distribution of mass. This is a demonstration of Wolff’s law (Wolff, 1893). While a detailed mechanical analysis of the tibia using finite element analysis is beyond the scope of this paper, we are confident that the utilised beam theory predicted the location of the null axis and peak strains in the cross section well. Estimation of the exact magnitude of peak strain as a function of load is not the focus of the current paper.

Early understandings of bone’s adaptive response suggested a range of strain levels which would not elicit an adaptive response, often referred to as the lazy zone (Carter, 1984; Huiskes et al., 1987). More recent studies have suggested that this region is non-existent in both animal models and in human tissue (Sugiyama et al., 2012; Ellman et al., 2013; Schulte et al., 2013; Christen et al., 2014). Likewise, in this work, a lazy zone is not observed. In line with previous studies, the results presented here suggest that no lazy zone exists in adaptive bone (re)modelling.

A limitation of this study is that the measurement technique does not determine if adaptation has occurred on the periosteal or endosteal surface, rather it only determines the net thickness change. Furthermore, the neurectomy performed on the mice removes habitual loading and induces some amount of resorption, with loading inducing additional bone formation subsequently. Due to this phenomenon, we are unable to quantitatively determine the amount of new bone material formed or resorbed on each surface, only the total difference after completion of the experiment. Longitudinal imaging would provide a significant benefit in this regard. Comparing a single limb at different time points, differences on both the endosteum and periosteum could be tracked to provide deeper insights into the mechanisms of bone adaptation.

Effects of loading on neurectomised vs. non-neurectomised mice were also not considered in the original study. While the left limb was left intact, performing a sciatic neurectomy on the right limb may have affected the gait of the mouse, potentially altering the habitual strains experienced in the healthy limb. Without such a control, it is difficult to answer several questions such as how bone adapts to mechanical loads from a standard habitual state (i.e., healthy gait), what are the bone loss effects of neurectomy, and what are the differences in adaptation response between healthy versus mechanically deficient (i.e., neurectomised) mice. Answering these questions would help to provide a more complete understanding of bone adaptation and should be investigated in future studies.

In this paper, we presented a novel image processing algorithm to measure cortical thickness of the mouse tibia loading model and compared the results across several loading magnitudes. We identified that discrete locations around the periosteum were shown to follow a quasi-linear cortical thickness adaptation response with increased loading, while points at areas of near-zero strain (i.e., neutral bending axis) experienced resorption regardless of loading magnitude; the correlation between strain and bone formation was shown to follow the adaptation principles of Wolff’s Law.

The ultimate purpose of animal adaptation studies is to derive mechanistic insights into the link between applied mechanical loads and the observed organ- or tissue-scale changes of (cortical) bone. The work conducted here has established a statistically significant association of mechanical loading and bone adaptation responses in discrete periosteal regions of cortical bone. The fact that these regions also experienced high compressive and tensile strains obtained from beam theory provides confidence that a mechanistic relationship exists between a particular mechanical quantity (such as principal strain, strain energy density, etc.) and the local cortical thickness changes. These findings may be useful in the development of treatments that aim to increase bone strength, informing specific mechanical loading routines that would provide targeted bone formation in areas of high fracture risk. Results we have obtained here will help develop novel bone adaption algorithms which are able to predict cortical thickness changes which is the scope of a future study.

## Data Availability Statement

The data analyzed in this study is subject to the following licenses/restrictions: Data used in this study is owned by the University of Bristol. Requests to access these datasets should be directed to PP, peter.pivonka@qut.edu.au.

## Ethics Statement

The animal study was reviewed and approved by University of Bristol.

## Author Contributions

CM: conceptualisation, development of algorithm to measure cortical thickness, beam theory analysis, collection of results, formal analysis, methodology, writing-original draft, and writing, review, and editing. ST: development of algorithm to measure cortical thickness, collection of preliminary results, and review and editing. EP: conceptualisation, beam theory analysis, data interpretation, and review and editing. SM: beam theory analysis and review and editing. PD and LM: data curation and review and editing. PP: conceptualisation, data curation, data analysis, funding acquisition, investigation, methodology, project administration, supervision, writing-original draft, and writing, review, and editing. All authors contributed to the article and approved the submitted version.

## Conflict of Interest

The authors declare that the research was conducted in the absence of any commercial or financial relationships that could be construed as a potential conflict of interest.
