# Supplementary material for: Cortical Thickness Adaptive Response to Mechanical Loading Depends on Periosteal Position and Varies Linearly With Loading Magnitude
Source: Front Bioeng Biotechnol. 2021 Jun 18;9:671606. doi: 10.3389/fbioe.2021.671606 (PMC8249932; doi:10.3389/fbioe.2021.671606)
Supplement: Supplementary file 1 [file Table_1.DOCX]

Supplementary Material

**
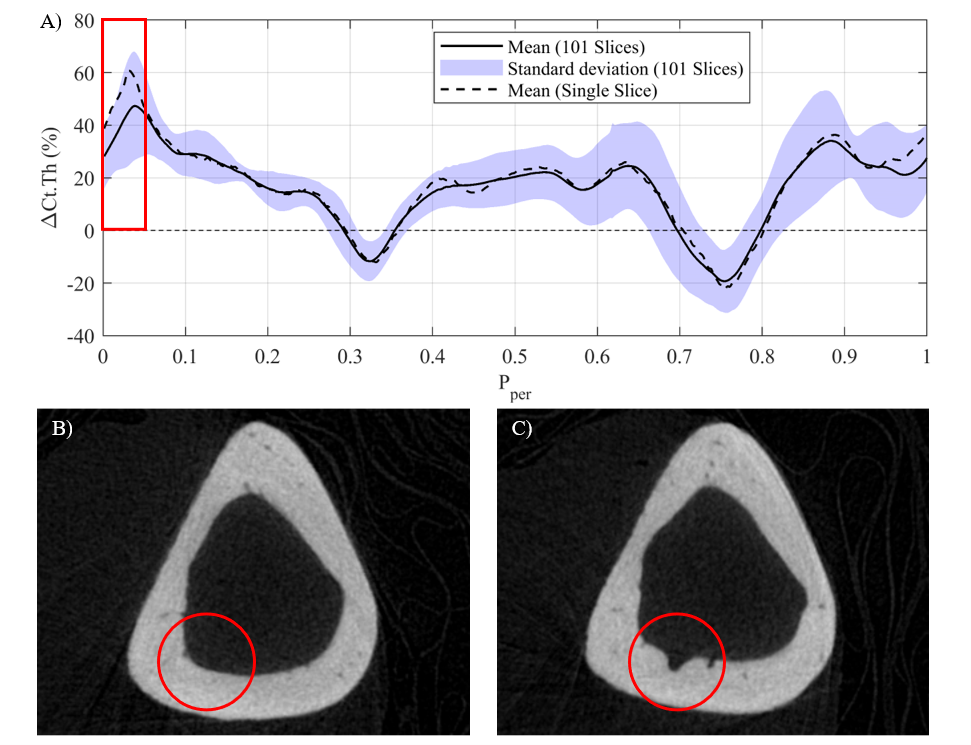
**

**Supplementary Figure 1.** Comparison of mean $\Delta$Ct.Th results between analysis of a single slice (dashed black line) and a representative average taken over 101 $\mu$CT slices (± 50 slices from the selected slice, measuring 482.75 $\mu$m) above and below the 50% region of the mouse tibia for a 12 N peak load ((A) solid black line). Mean $\Delta$Ct.Th measurements obtained from a single slice closely match the mean results of the representative slice for the majority of the periosteal surface. The most notable variation occurs between 0 < L_per_ < 0.05 (A, red box), where the mean $\Delta$Ct.Th of the single slice was observed to be approximately equal to the mean $\Delta$Ct.Th plus one standard deviation of the representative slice. This variation stems from the absence (B) or presence (C) of blood vessel channels, circled in red. Taking a representative average across a large region of the tibia smooths out these channels, therefore affecting the observed growth percentage.
